# Unraveling the role of cumulative triglyceride-total cholesterol-body weight index in stroke development: evidence from the CHARLS cohort

**DOI:** 10.3389/fmed.2025.1616520

**Published:** 2025-07-10

**Authors:** Huang Luwen, Zhang Yunwei, Xu Lei, Li Linlin, Yu Ming

**Affiliations:** Department of Neurology, Suining Central Hospital, Suining, Sichuan, China

**Keywords:** triglyceride-total cholesterol-body weight index, stroke, non-linearly relationship, mediation analysis, CHARLS

## Abstract

**Background:**

This study investigated the association between the cumulative triglyceride-total cholesterol-body weight index (TCBI) and the risk of stroke among middle-aged and older adults, focusing on hypertension as a potential mediator.

**Methods:**

Data from 5,598 participants aged ≥ 45 years in the China Health and Retirement Longitudinal Study were analyzed over a median follow-up of 57.2 months. CumTCBI was calculated as ((TCBI_(2011)_ + TCBI_(2015)_)/2) × (2015–2011). The risk of stroke was the primary outcome. Cox proportional hazards models and restricted cubic splines were used to examine the association between CumTCBI and stroke risk. Mediation analysis investigated the role of hypertension as a potential mediator of the association between CumTCBI and stroke risk.

**Results:**

During the follow-up period, 480 (8.93%) participants experienced stroke. The fully adjusted CumTCBI was significantly associated with stroke (HR per 1 SD = 1.166). A non-linearly association was observed, with stroke risk increasing when CumTCBI was below 12.639 (HR per 100 units = 1.166, *P* = 0.002) and remaining stable beyond this threshold (*P* = 0.356). Additionally, hypertension mediated 27.4% of the association.

**Conclusion:**

CumTCBI is non-linearly associated with stroke risk, partially mediated by hypertension. Managing both metabolic status and hyperternsion may reduce stroke risk in aging populations.

## 1 Introduction

Stroke remains one of the leading causes of death and disability worldwide, particularly among middle-aged and older adults, highlighting the importance of addressing its modifiable risk factors (1, 2). According to recent global statistics, approximately 11.9 million strokes occur annually worldwide, and stroke-related Disability-Adjusted Life Years (DALYs) total 160.5 million, underscoring the substantial burden on global health systems (3). Identifying modifiable risk factors is critical for developing effective prevention and intervention strategies. Individual biomarkers such as body mass index (BMI), waist-to-hip ratio, total cholesterol (TC) (4, 5), and triglycerides (TG) (6) have been linked to stroke risk. However, we propose that integrating multiple metabolic indices, such as the Triglyceride-Total Cholesterol-Body Weight Index (TCBI), may offer a more comprehensive approach to stroke risk assessment.

TCBI (Triglyceride-cholesterol-body-weight index) is a composite index that incorporates TG, TC, and body weight (BW). The formula for TCBI is: TCBI = TG (mg/dL) × TC (mg/dL) × BW (kg)/1,000. It is important to note that TCBI is a unitless index, offering a standardized measure of metabolic health. Elevated TCBI has been linked to increased risk of stroke-associated pneumonia in patients with acute ischemic stroke (7), as well as various adverse cardiovascular disease (CVD) outcomes (7–10). However, evidence on the association between TCBI and the risk of stroke or CVD remains inconsistent. For example, Rezaee et al. (10) reported a positive association between TCBI and CVD mortality (10), whereas another cross-sectional study in hypertensive individuals found an inverse association with stroke risk (8). The limited population scope and study design may partially explain these discrepancies. Additionally, most existing studies relied on single-timepoint TCBI measurements, which may not capture long-term metabolic burden. To address this limitation, we introduced the cumulative TCBI (CumTCBI), calculated as the time-weighted average of TCBI values from 2011 to 2015. This approach better reflects long-term metabolic exposure and reduces short-term variability. Cumulative exposure is considered an important factor in assessing metabolic risk, and recent studies have also shown that cumulative indices help improve the prediction of chronic disease risk (11, 12).

To bridge these gaps, this study utilizes data from the China Health and Retirement Longitudinal Study (CHARLS) to investigate the longitudinal association between CumTCBI and the risk of stroke. By elucidating these relationships, our findings may offer scientific evidence to support targeted interventions aimed at addressing dyslipidemia and weight—key components captured by TCBI—thereby ultimately mitigating stroke risk in middle-aged and older adults.

## 2 Materials and methods

### 2.1 Data source and study population

The data used in this study were obtained from CHARLS, a nationally representative longitudinal cohort study focusing on individuals aged 45 years and older. This prospective cohort study began with the first nationwide survey conducted in 2011 (Wave 1), followed by assessments in 2013, 2015, 2018, and 2020 (Waves 2, 3, 4, and 5, respectively) (13). Blood samples were collected from participants in Waves 1 and 3.

**FIGURE 1:**
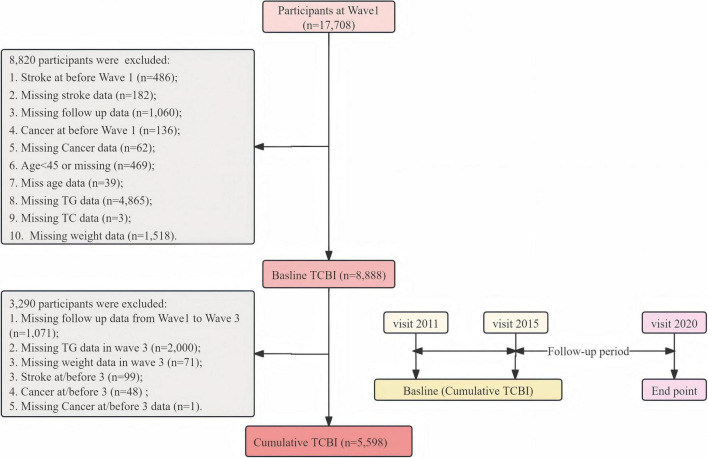
Flowchart of study populations.

To evaluate the correlation between the CumTCBI score and stroke risk, we selected 5,598 participants with complete data on TG, TC, and weight from both the first and third waves and no history of stroke during this period. Additionally, we excluded participants with missing follow-up data and a history of cancer. The follow-up period in this study commenced with the last baseline data point (2015) and continued through 2020. Due to limitations within the research team, there were slight variations of a few months in the start and end times of follow-up for certain participants. A detailed outline of the participant inclusion criteria is presented in Figure 1.

The CHARLS study involving human participants was approved by the Biomedical Ethics Review Board of Peking University (IRB00001052–11015) (13). Written informed consent was obtained from all participants. The study followed the Reporting of Observational Studies in Epidemiology guidelines for observational studies (14).

### 2.2 Exposure variables

To approximate long-term metabolic exposure, we calculated cumulative values (e.g., CumTCBI and other laboratory parameters) using the time-weighted average of two available time points (2011 and 2015). This method assumes a linear change between measurements and has been widely adopted in epidemiological studies where continuous data are not available. Similar cumulative exposure models have been applied to LDL-C (15), remnant cholesterol (16), and hs-CRP in prior research (16), demonstrating reasonable validity in estimating chronic metabolic burden.

The variables extracted from the CHARLS include the following:

(1) General clinical characteristics: age, sex, residence, education, marital status, blood pressure, CumBMI, smoking status, and drinking status. Unlike other cumulative variables, age is a natural, irreversible process that increases over time. Therefore, in this study, age was treated as a fixed baseline variable, defined as the participant’s age in 2011, and was not accumulated. Instead, it was included as a control variable in the statistical analysis.
(2) Comorbidities: hypertension, dyslipidemia, heart disease, diabetes, liver disease, and kidney disease. Hypertension was defined as having a systolic blood pressure ≥ 140 mmHg or diastolic blood pressure ≥ 90 mmHg (17). Participants with a self-reported history of hypertension or those using antihypertensive medication were also classified as hypertensive (18). Blood pressure measurements were taken after the participant rested for at least 10 min in a sitting position. Three measurements were recorded at 5-min intervals, and the average of the three readings was used for analysis. Participants were diagnosed with diabetes if previously diagnosed by a physician or if their examination showed fasting blood glucose (FBG) ≥ 7.0 mmol/L (126 mg/dL) or HbA1c ≥ 6.5%, in accordance with WHO and ADA guidelines (19). Dyslipidemia was defined as a TC/high-density lipoprotein cholesterol (HDL-C) ratio > 5.0 or self-reported dyslipidemia (19). Participants were also considered to have heart disease, liver disease, or cirrhosis if previously diagnosed by a physician, as self-reported (20). Chronic kidney disease was diagnosed based on self-report of a physician diagnosis (20).
(3) Laboratory parameters: cumulative laboratory parameters—including CumFBG, hemoglobin A1c (CumHbA1c), CumTG, CumTC, CumHDL-C, low-density lipoprotein cholesterol (CumLDL-C), serum creatinine (CumScr), and uric acid (CumUA)—were calculated as the means of measurements from 2011 to 2015 ((value_(2011)_ + value_(2015)_)/2) × (2015–2011).

The TCBI for each participant was calculated via the following formula: TCBI = TG (mg/dL) × TC (mg/dL) × BW (kg)/1,000 (10). CumTCBI is determined as the time-weighted average of the TCBI values from 2011 to 2015, calculated as: CumTCBI = ((TCBI_(2011)_ + TCBI_(2015)_)/2) × (2015–2011).

### 2.3 Outcome

The primary outcome was incident stroke, identified through self-reported physician diagnosis (“Have you ever been diagnosed with stroke by a doctor?”) (21, 22). Stroke onset was assigned to the interval between the last stroke-free interview and the first interview reporting stroke. For participants without incident stroke, follow-up ended at their last available interview.

### 2.4 Missing variables

The distribution of variables with missing data is shown in Supplementary Table 1, with a low proportion of missing values (no variable had more than 10% missing data). To address this, multiple imputation with 5 replications was applied, ensuring the retention of the maximum sample size and enhancing the precision and robustness of the analysis.

### 2.5 Statistical analysis

Baseline characteristics are presented as the means ± standard deviations (SDs) or medians (IQRs) for continuous variables and percentages for categorical variables. Cox proportional hazard models were used to evaluate the association between CumTCBI and the risk of stroke, adjusting for potential confounders. The crude model was unadjusted. Model 1 was adjusted for age and sex. Model 2 included the adjustments in Model 1, with additional adjustments for residence, education, smoking, and drinking status. Model 3 was further adjusted for all covariates in Model 2, as well as hypertension, heart disease, diabetes, liver disease, kidney disease, CumFBG, CumHbA1c, CumLDL-C, CumScr, and CumUA.

The study employed the Cox model with restricted cubic splines (RCS) to assess the dose-response relationship between CumTCBI and stroke risk. The data were fitted using both 3 and 4 knots. Knot placement was determined based on the data distribution and the model’s flexibility. In the 3-knot model, knots were placed at the 10th, 50th, and 90th percentiles of the data, while in the 4-knot model, they were positioned at the 25th, 50th, 75th, and 90th percentiles. Moreover, to determine the optimal threshold for the two-piecewise linear regression model, we used log-likelihood to compare the fit of different threshold values and identified the threshold in the non-linearly relationship between CumTCBI and stroke risk. To enhance data visualization, the CumTCBI values were divided by 100 in the RCS model and threshold effect analysis. This transformation only affects the graphical presentation and threshold effect analysis, while no scaling was applied to the CumTCBI values in other analyses.

Stratified analyses were conducted to assess the robustness of the findings. Furthermore, a mediation analysis was conducted to evaluate whether factors (hypertension, diabetes, WBC, hs-CRP) mediate the association between CumTCBI and stroke. The average causal mediation effect, average direct effect, total effect, and the proportion mediated were estimated. Confidence intervals were calculated using a non-parametric bootstrap approach with 1,000 resamples and the percentile method. The analysis was adjusted for the covariates included in Model 3 of the Cox regression, except for the mediator variables. It was conducted under standard assumptions, including the absence of unmeasured confounding.

### 2.6 Sensitivity analysis

A series of sensitivity analyses were performed to increase the robustness of the study findings. First, a Cox regression model was applied after excluding participants with missing data. To address the missing data, multiple imputation was performed using 5 replications and the Markov-chain Monte Carlo method within the SAS MI procedure. Additionally, a well-matched cohort was established via 1:1 propensity score matching (PSM) with nearest-neighbor matching, no replacement, and a caliper width of 0.01 of the standard deviation of the logit of the propensity score. The balance between groups (Q1-Q2 vs. Q3-Q4) was assessed via the standardized mean difference (SMD), with an SMD below 0.10 indicating an acceptable balance.

All analyses were conducted via R software (version 3.3.2, The R Foundation)^1^ and Free Statistics software (version 1.7). A two-tailed *P* < 0.05 was considered statistically significant.

## 3 Results

### 3.1 Baseline characteristics

A comparison of the characteristics of 5,598 participants revealed significant differences across quartiles of the CumTCBI in terms of sex, age, residence, marital status, blood pressure, CumBMI, smoking, dyslipidemia, hypertension, heart disease, diabetes, and laboratory parameters (all *P* < 0.001) (Table 1). The proportion of urban residents increased across the quartiles, reaching 40.79% in Q4. The mean age showed a decreasing trend, from 60.06 years in Q1 to 57.18 years in Q4 (*P* < 0.001). With respect to comorbidities, the prevalence of dyslipidemia, hypertension, heart disease, and diabetes increased significantly with increasing CumTCBI quartiles (all *P* < 0.001). The laboratory parameters revealed notable increases in the CumFBG, CumTG, and CumTC levels with increasing CumTCBI, whereas the CumHDL-c level significantly decreased (*P* < 0.001). Additionally, the median CumTCBI index was significantly greater in the stroke group (1473.6) than in the non-stroke group (1243.9) (*P* < 0.001), suggesting a potential association between the CumTCBI score and stroke risk (Supplementary Table 2).

**TABLE 1:** Baseline characteristics of individuals classified by quartiles of the CumTCBI.

Characteristics	Overall	Quartiles of the CumTCBI				
		Q1 (< 832)	Q2 (833–1261)	Q3 (1262–2018)	Q4 (> 2019)	*P*-value
n	5,598	1,400	1,399	1,399	1,400	
Sex (female)	3,096 (55.31%)	661 (47.21%)	768 (54.90%)	838 (59.90%)	829 (59.21%)	< 0.001
Age, years	58.67 ± 8.52	60.06 ± 9.08	59.23 ± 8.51	58.22 ± 8.31	57.18 ± 7.84	<0.001
Residence (Urban)	1,829 (32.67%)	344 (24.57%)	442 (31.59%)	472 (33.74%)	571 (40.79%)	< 0.001
Education						0.364
Elementary school or below	2,683 (47.95%)	729 (52.11%)	699 (50.00%)	651 (46.53%)	604 (43.17%)	
Middle school	2,393 (42.77%)	564 (40.31%)	572 (40.92%)	617 (44.10%)	640 (45.75%)	
College or above	519 (9.28%)	106 (7.58%)	127 (9.08%)	131 (9.36%)	155 (11.08%)	
Current married	5,015 (89.59%)	1,218 (87.00%)	1,238 (88.49%)	1,270 (90.78%)	1,289 (92.07%)	<0.001
SBP, mmHg	128.32 ± 20.75	123.39 ± 20.40	127.82 ± 20.04	129.65 ± 20.80	132.41 ± 20.74	< 0.001
DBP, mmHg	75.00 ± 12.00	71.30 ± 11.42	74.54 ± 11.34	75.64 ± 11.92	78.52 ± 12.20	< 0.001
CumBMI, kg/m^2^	24.37 ± 34.68	21.55 ± 13.04	23.31 ± 15.07	26.57 ± 66.23	26.05 ± 3.87	<0.001
Smoking						< 0.001
Never	3,489 (62.33%)	792 (56.57%)	866 (61.90%)	926 (66.19%)	905 (64.64%)	
Former	446 (7.97%)	102 (7.29%)	101 (7.22%)	106 (7.58%)	137 (9.79%)	
Current	1,663 (29.71%)	506 (36.14%)	432 (30.88%)	367 (26.23%)	358 (25.57%)	
Drinking						0.015
Never	3,325 (59.43%)	780 (55.75%)	829 (59.30%)	865 (61.87%)	851 (60.79%)	
Former	429 (7.67%)	114 (8.15%)	95 (6.80%)	107 (7.65%)	113 (8.07%)	
Current	1,841 (32.90%)	505 (36.10%)	474 (33.91%)	426 (30.47%)	436 (31.14%)	
**Comorbidities**						
Dyslipidemia	511 (9.31%)	41 (2.97%)	92 (6.69%)	137 (10.01%)	241 (17.62%)	< 0.001
Hypertension	2,582 (46.23%)	465 (33.31%)	608 (43.55%)	695 (49.82%)	814 (58.23%)	< 0.001
Heart disease	617 (11.05%)	105 (7.52%)	134 (9.60%)	173 (12.43%)	205 (14.66%)	< 0.001
Diabetes	779 (14.00%)	120 (8.60%)	139 (9.98%)	184 (13.28%)	336 (24.17%)	< 0.001
Liver disease	175 (3.14%)	50 (3.58%)	49 (3.52%)	37 (2.66%)	39 (2.80%)	0.370
Kidney disease	302 (5.42%)	78 (5.58%)	77 (5.53%)	71 (5.09%)	76 (5.46%)	0.940
**Laboratory parameters**						
CumFBG, mg/dL	106.57 ± 30.00	99.01 ± 20.00	102.67 ± 26.90	107.18 ± 30.76	117.42 ± 36.61	<0.001
CumHbA1c, %	5.63 ± 0.81	5.47 ± 0.60	5.54 ± 0.69	5.65 ± 0.87	5.88 ± 0.98	<0.001
CumTG, mg/dL	134.71 ± 113.09	69.68 ± 22.28	95.74 ± 29.47	130.47 ± 44.40	242.92 ± 174.20	<0.001
CumTC, mg/dL	193.87 ± 38.58	171.30 ± 30.12	188.76 ± 31.66	198.19 ± 33.32	217.22 ± 42.84	<0.001
CumHDL-C, mg/dL	51.40 ± 12.48	57.28 ± 13.28	54.14 ± 12.09	49.75 ± 10.62	44.43 ± 9.72	<0.001
CumLDL-C, mg/dL	109.97 ± 28.63	95.07 ± 21.56	110.72 ± 23.46	117.18 ± 26.75	116.98 ± 35.07	<0.001
CumScr, mg/dL	0.77 ± 0.18	0.75 ± 0.18	0.76 ± 0.17	0.77 ± 0.17	0.78 ± 0.19	<0.001
CumUA, mg/dL	4.37 ± 1.21	4.14 ± 1.13	4.23 ± 1.17	4.37 ± 1.19	4.72 ± 1.26	<0.001
CumTCBI	1,684.456 ± 1,626.416	623.82 ± 136.46	1,036.12 ± 122.56	1,591.61 ± 213.83	3,485.75 ± 2,387.84	<0.001

SBP, systolic blood pressure; DBP, diastolic blood pressure; CumBMI, cumulative body mass index; CumFBG, cumulative fasting blood glucose; CumHbA1c, cumulative glycosylated hemoglobin A1c; CumTG, cumulative triglyceride; CumTC, cumulative total cholesterol; CumHDL-c, cumulative high-density lipoprotein cholesterol; CumLDL-c, cumulative low-density lipoprotein cholesterol; CumSCr, cumulative serum Creatinine; CumUA, cumulative uric acid; CumTCBI, cumulative triglyceride Cholesterol Body-weight Index.

### 3.2 Associations between the CumTCBI and the risk of stroke

**FIGURE 2:**
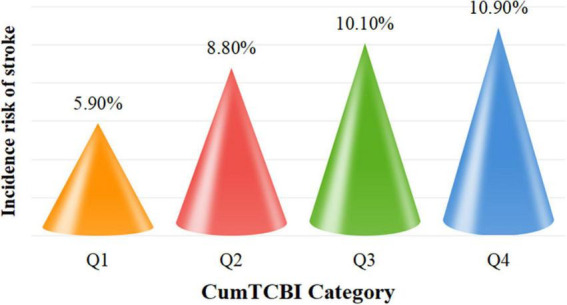
Incidence rates of stroke categorized by quartiles of the cumulative. CumTCBI. Q1, Quartile 1; Q2, Quartile 2; Q3, Quartile 3; Q4, Quartile 4.

**FIGURE 3:**
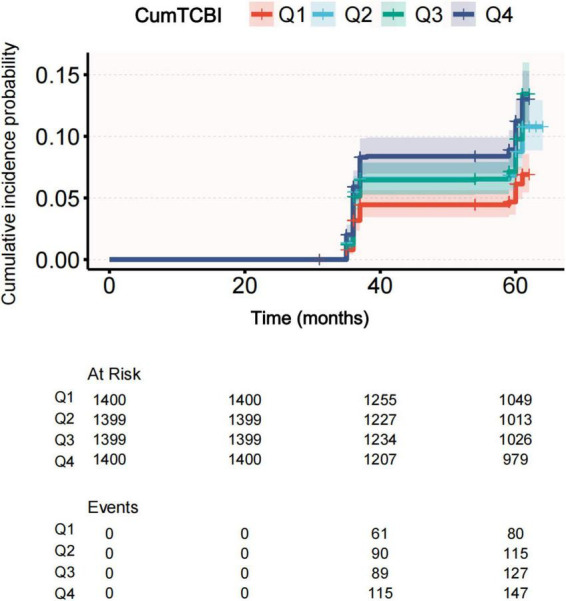
Cumulative incidence of stroke across quartiles of CumTCBI.

The incidence rate of stroke increased across quartiles of CumTCBI, with rates of 83 (5.90%) in Q1, 123 (8.80%) in Q2, 141 (10.10%) in Q3, and 153 (10.90%) in Q4 (Figure 2; Table 1). To further investigate this relationship, we analyzed the association between CumTCBI and the risk of stroke using both quartile-based and continuous variables. The crude HR was significantly associated with the risk of stroke, with HRs of 1.511 (95% CI: 1.144, 1.996) for Q2, 1.730 (95% CI: 1.319, 2.270) for Q3, and 1.924 (95% CI: 1.473, 2.513) for Q4, with a trend *P* < 0.001 (Table 2). In Model III, which was adjusted for extensive covariates, the HR for Q4 compared with Q1 was 1.668 (95% CI: 1.24, 2.243), indicating a persistently elevated risk. When analyzed as a continuous variable, each 1-SD increase in CumTCBI was associated with a 1.094-fold increase in stroke risk in the crude model (95% CI: 1.040, 1.150), and the association remained statistically significant after adjusting for multiple confounders across different models. The cumulative incidence of stroke increased across quartiles of CumTCBI over 60 months (Figure 3). The risk of stroke was lowest in Q1 and highest in Q4, with Q2, and Q3 showing increasing trends.

**TABLE 2:** Association between the CumTCBI and the risk of stroke.

CumTCBI	Quartiles					Continuous
	Q1 (< 833)	Q2 (833–1262)	Q3 (1263–2019)	Q4 (> 2019)	P for trend	Per 1 SD increase
Median	637.2	1,030	1,570	2,810.3		
Cases, n (%)	83 (5.9%)	123 (8.8%)	141 (10.1%)	153 (10.9%)	–	–
Crude, HR (95% CI)	Reference	1.511 (1.144, 1.996)	1.73 (1.319, 2.27)	1.924 (1.473, 2.513)	< 0.001	1.094 (1.04, 1.15)
Model 1, HR (95% CI)	Reference	1.562 (1.182, 2.065)	1.847 (1.406, 2.427)	2.144 (1.636, 2.81)	< 0.001	1.12 (1.06, 1.17)
Model 2, HR (95% CI)	Reference	1.559 (1.179, 2.062)	1.859 (1.413, 2.444)	2.159 (1.643, 2.837)	< 0.001	1.119 (1.065, 1.175)
Model 3, HR (95% CI)	Reference	1.42 (1.068, 1.889)	1.544 (1.158, 2.058)	1.668 (1.24, 2.243)	0.001	1.166 (1.064, 1.278)

Crude model adjusts for: none. Model I adjusts for sex and age. Model II adjusts for Model I, marital status, residence, education, smoking, and drinking. Model III adjusts for Model II, hypertension, heart disease, diabetes, liver disease, kidney disease, CumFBG, CumHbA1c, CumLDL-C, CumScr, and CumUA. CI, confidence interval; HR, hazard ratio.

**FIGURE 4:**
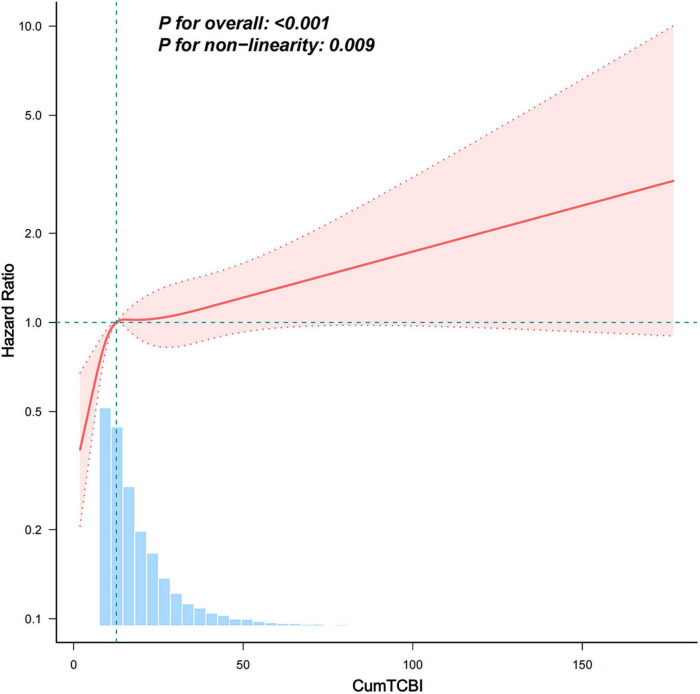
Association between the CumTCBI (per 100 scaled) and the risk of stroke. The model was adjusted for gender, age, marital status, residence, education, smoking, drinking, hypertension, heart disease, diabetes, liver disease, kidney disease, CumFBG, CumHbA1c, CumLDL-C, CumScr, and CumUA. The RCS model was used with 3 knots to account for non-linearity in the relationship.

Both the RCS and threshold effect analyses were based on CumTCBI values scaled by 100. The analysis revealed a non-linearly association between CumTCBI and stroke risk for both the 3-knot (Supplementary Figure 1) and 4-knot models (Figure 4). Further, a threshold analysis identified an inflection point at 12.639 (95% CI: 11.517, 13.761) (per 100 scale) (Table 3). Below this threshold, each 100-unit increase in CumTCBI was associated with a 1.108-fold increase in stroke risk (95% CI: 1.04, 1.18, *P* = 0.002). However, beyond this point, the association became non-significant (HR = 1.004, 95% CI: 0.995, 1.013, *P* = 0.356).

**TABLE 3:** Threshold-effect analysis of the relationship between the CumTCBI (per 100 scaled) and the risk of stroke.

Models	Per-100 unit increase	
	HR (95%CI)	*P*-value
**Model I**		
One line effect	1.009 (1.004, 1.015)	0.001
**Model II**		
Turning point (K)	12.639 (11.517,13.761)	
CumTCBI < K	1.108 (1.04, 1.18)	0.002
CumTCBI ≥ K	1.004 (0.995, 1.013)	0.356
*P*-value for LRT test*		0.006

Model I, linear analysis; Model II, non-linearly analysis. Adjusted for sex, age, marital status, residence, education, smoking, drinking, hypertension, heart disease, diabetes, liver disease, kidney disease, CumFBG, CumHbA1c, CumLDL-C, CumScr, and CumUA.

*P < 0.05 indicates that Model II is significantly different from Model I. LRT, logarithm likelihood ratio test.

### 3.3 Subgroup analysis

**FIGURE 5:**
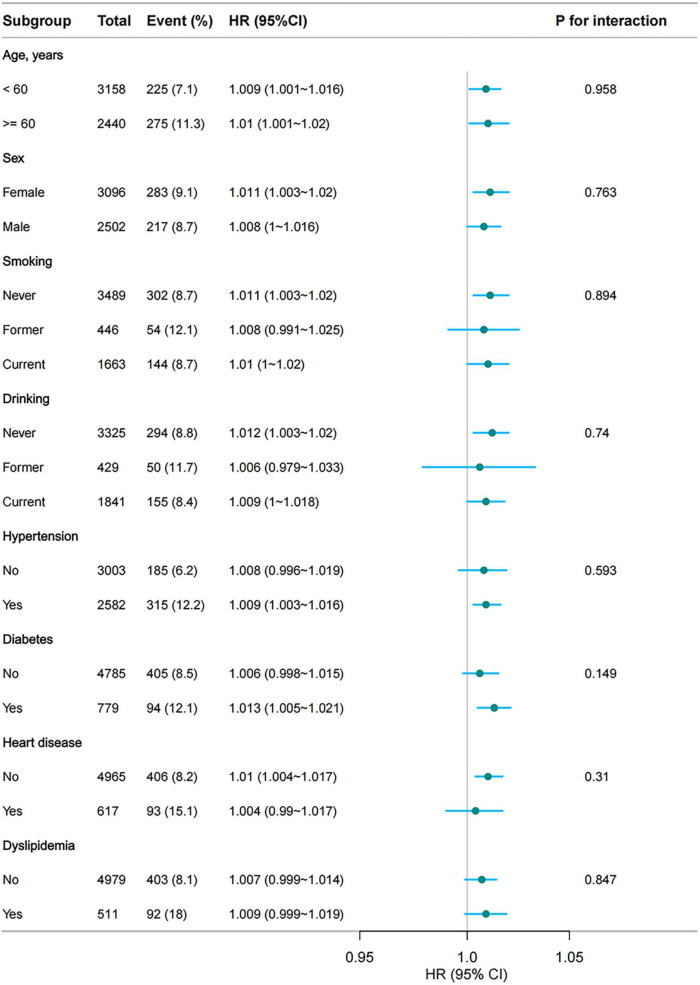
Subgroup analyses of the association between CumTCBI (per 100 scaled) and the risk of stroke. The model was adjusted for gender, age, marital status, residence, education, smoking, drinking, hypertension, dyslipidemia, heart disease, diabetes, liver disease, kidney disease, CumFBG, CumHbA1c, CumLDL-C, CumScr, and CumUA.

Subgroup analysis revealed that for every 100-unit increase in the CumTCBI, the HR for stroke remained stable across all subgroups, with no statistically significant interaction effects (*P* > 0.05) (Figure 5). Age, sex, smoking, drinking, hypertension, heart disease, and dyslipidemia did not significantly modify this association. Although the HR was slightly greater in individuals with diabetes (1.013, 95% CI: 1.005, 1.021) than in non-diabetic individuals (HR = 1.006, 95% CI: 0.998, 1.015), the interaction effect was not significant (*P* = 0.149). These findings indicate that the association between TCBI and stroke risk is consistent across different subgroups.

### 3.4 Mediation analysis

**FIGURE 6:**
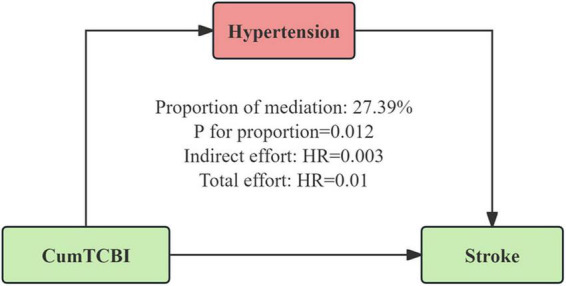
Hypertension as a mediator of the relationship between CumTCBI and the risk of stroke. The model was adjusted for gender, age, marital status, residence, education, smoking, drinking, hypertension, heart disease, diabetes, liver disease, kidney disease, CumFBG, CumHbA1c, CumLDL-C, CumScr, and CumUA.

The mediation analysis revealed that hypertension partially mediated the association between CumTCBI and the risk of stroke (Figure 6; Table 4), with a mediation proportion of 27.4% (*P* = 0.012). The indirect effect of CumTCBI on stroke through hypertension was HR = 0.003, while the total effect was HR = 0.01. Additionally, CumTCBI mediated 4.3% of the association between hypertension and the risk of stroke (Table 5). Other factors could not adjust the relationship between CumTCBI and the risk of stroke (Table 6).

**TABLE 4:** Mediation analysis of the effect of CumTCBI on the risk of stroke through hypertension.

Effect type	Estimate	95% CI	*P*-value
Total effect	0.01	(0.003, 0.016)	0.012
Direct effect	0.007	(0.001, 0.013)	0.032
Indirect effect	0.003	(0.001, 0.003)	< 0.001
Proportion mediated	0.274	(0.075, 0.648)	0.012

Adjusted for gender, age, marital status, residence, education, smoking, drinking, heart disease, diabetes, liver disease, kidney disease, CumFBG, CumHbA1c, CumLDL-C, CumScr, and CumUA.

**TABLE 5:** Mediation analysis of the effect of hypertension on the risk of stroke through CumTCBI.

Effect Type	Estimate	95% CI	*P*-value
Total effect	0.04	(0.023, 0.056)	<0.001
Direct effect	0.038	(0.022, 0.054)	<0.001
Indirect effect	0.002	(0.0002, 0.003)	0.026
Proportion mediated	0.043	(0.005, 0.105)	0.026

Adjusted for gender, age, marital status, residence, education, smoking, drinking, heart disease, diabetes, liver disease, kidney disease, CumFBG, CumHbA1c, CumLDL-C, CumScr, and CumUA.

**TABLE 6:** Mediation analysis of the associations between CumTCBI and stroke across different factors.

Mediators	Associations, HR (95% CI)	
	Total	Direct
Diabetes	0.007 (0.001, 0.013)	0.008 (0.001, 0.013)
FBG	0.008 (0.002, 0.014)	0.007 (0.001, 0.014)
WBC	0.008 (0.001, 0.015)	0.008 (0.0011, 0.0144)
hs-CRP	0.009 (0.003, 0.015)	0.009 (0.0027, 0.151)

Adjusted for gender, age, marital status, residence, education, smoking, drinking, heart disease, diabetes, liver disease, kidney disease, CumFBG, CumHbA1c, CumLDL-C, CumScr, and CumUA (except for the mediator variables).

### 3.5 Sensitivity analysis

To assess the robustness of the study findings, a series of sensitivity analyses was conducted. First, after individuals with missing values were excluded, the associations between CumTCBI quartiles and stroke risk remained significant (Supplementary Table 3, P for trend = 0.001). Second, after missing values were handled via multiple imputations, the associations remained significant (Table 7, P for trend < 0.001). Furthermore, after 1:1 propensity score matching, the high CumTCBI group (Q3–Q4) had a significantly greater stroke risk than the low CumTCBI group did (Q1–Q2) (Table 8, HR = 1.462, 95%CI: 1.186, 1.801, *P* = 0.006). Additionally, each SD increase in the CumTCBI was significantly associated with increased stroke risk (HR = 1.201, 95% CI: 1.064, 1.399, *P* = 0.004). After propensity score matching, the SMD of each covariate between the two groups was significantly reduced, indicating effective matching (Supplementary Table 4). Overall, these sensitivity analysis results confirm the robustness of the association between the CumTCBI and stroke risk.

**TABLE 7:** Association between the CumTCBI and the risk of stroke after multiple imputations.

CumTCBI	Quartiles					Continuous
	Q1	Q2	Q3	Q4	P for trend	Per 1 SD increase
Median	637.2	1,030	1,570	2,810.3		
Cases, n (%)	83 (5.9%)	123 (8.8%)	141 (10.1%)	153 (10.9%)	–	–
Crude, HR (95% CI)	Reference	1.511 (1.144, 1.996)	1.73 (1.319, 2.27)	1.924 (1.473, 2.513)	< 0.001	1.006 (1.002, 1.009)
Model 1, HR (95% CI)	Reference	1.562 (1.182, 2.065)	1.847 (1.406, 2.427)	2.144 (1.636, 2.81)	< 0.001	1.257 (1.164, 1.358)
Model 2, HR (95% CI)	Reference	1.57 (1.188, 2.076)	1.859 (1.413, 2.445)	2.159 (1.643, 2.837)	< 0.001	1.117 (1.065, 1.172)
Model 3, HR (95% CI)	Reference	1.442 (1.085, 1.916)	1.596 (1.198, 2.126)	1.691 (1.258, 2.274)	< 0.001	1.068 (1.001, 1.141)

Crude model adjusts for: none. Model I adjust for sex and age. Model II adjusts for Model I, marital status, residence, education, smoking, and drinking. Model III adjusts for Model II, hypertension, heart disease, diabetes, liver disease, kidney disease, CumFBG, CumHbA1c, CumLDL-C, CumScr, CumUA. CI, confidence interval; HR, hazard ratio.

**TABLE 8:** Association between the cumTCBI and the risk of stroke after 1:1 propensity score matching.

CumTCBI	Cases, n (%)	HR (95% CI) *	*P*-value
**Categories**			
Quartile 1–2	133 (7.8)	Reference	–
Quartile 3–4	175 (10.3)	1.462 (1.186, 1.801)	0.006
**Continuous**			
Per 1 SD increase	–	1.201 (1.064, 1.399)	0.004

The HR with their 95% CI for univariable logistic regression model were presented since no further covariate adjustments were required after 1:1 propensity score matching (PSM).

## 4 Discussion

This study is the first to investigate the association between CumTCBI and the risk of stroke in middle-aged and older adults using a nationally representative sample from CHARLS. Our findings demonstrated a non-linearly positive correlation with the risk of stroke, providing preliminary evidence for the potential use of the TCBI as a tool for predicting stroke in this population. Additionally, hypertension was identified as a mediator in this association, suggesting that TCBI may influence stroke risk through its impact on blood pressure regulation. This finding offers insight into a potential mechanism linking TCBI to stroke development.

The TCBI is a newly developed, simple nutritional index that integrates serum TG, TC, and BW (9). As a composite marker, it reflects both nutritional status and metabolic reserves, making it a useful tool for assessing health risks associated with malnutrition and metabolic disorders (23, 24). Compared with traditional nutritional indices such as the Geriatric Nutritional Risk Index, Controlling Nutritional Status, and Prognostic Nutritional Index, the TCBI is more accessible and easier to calculate. It relies on routinely measured laboratory parameters, making it highly practical for clinical application (25, 26). Recent studies suggest that the TCBI may serve as an important prognostic marker in critically ill cardiac patients, including those with acute decompensated heart failure (24), chronic heart failure (27), and transcatheter aortic valve replacement (28). Furthermore, the TCBI has been explored as a potential predictor of adverse outcomes in diabetic nephropathy (29), all-cause and cardiovascular mortality in the general population (23), and even cognitive impairment (30). A study by Ishiwata et al. reported that higher TCBI levels may reflect better overall metabolic health and resilience, potentially contributing to a lower risk of mortality (23).

However, studies investigating the relationship between TCBI and the risk of stroke are limited. A study on TCBI and adverse functional outcomes in acute ischemic stroke patients revealed that lower TCBI levels were associated with poor prognosis (31). Similarly, Yu Feng et al. reported a greater risk of stroke-associated pneumonia in patients with low TCBI (7). However, these studies focused on stroke prognosis, whereas our study examined the association between CumTCBI and the risk of stroke in the general population, highlighting the potential role of TCBI in primary stroke prevention. Additionally, the China H-type Hypertension Registry Study revealed a linear inverse relationship between the baseline TCBI and the risk of stroke in hypertensive individuals (8). In contrast, our study revealed a positive association between the CumTCBI and the risk of stroke. Notably, this relationship remained consistent across all subgroups, including age, sex, smoking status, drinking status, and the prevalence of hypertension, diabetes, heart disease, or dyslipidemia, suggesting that the observed association is robust and independent of these factors. Furthermore, a non-linearly association between CumTCBI and stroke risk, with a plateau effect, was observed. In our study, 49.91% of participants had CumTCBI/100 values above the 12.639 threshold. Below this threshold, each 100-unit increase in CumTCBI was significantly associated with an increased stroke risk, but beyond this point, the association became non-significant. This suggests that for individuals with lower CumTCBI, increases in CumTCBI are strongly linked to stroke risk, while for those with higher values, the effect diminishes. This may be due to other risk factors, such as hypertension, diabetes, or vascular pathology, becoming more dominant beyond the threshold. Further exploration of these mechanisms is needed to better understand stroke risk. More aggressive control of CumTCBI is recommended for individuals with values below 12.639 to reduce stroke risk.

The mechanisms underlying the relationship between TCBI and stroke risk are complex and not yet fully understood. However, several potential mechanisms may help explain this association. First, our study identified hypertension as a mediator in the relationship between TCBI and stroke risk. Elevated TCBI reflects dysregulated lipid metabolism, typically associated with hypercholesterolemia and hypertriglyceridemia. These metabolic abnormalities contribute to the deposition of LDL-C in the arterial intima, thereby promoting the formation of atherosclerotic plaques (32–34). Atherosclerosis further leads to arterial stiffening, luminal narrowing, and the loss of vascular elasticity, disrupting blood pressure regulation (35–37). This process may activate the sympathetic nervous system and the renin-angiotensin-aldosterone system, both of which are known to raise blood pressure, exacerbating hypertension (38). As hypertension persists, endothelial dysfunction and inflammatory responses are aggravated, forming a vicious cycle that increases stroke risk (39). Therefore, hypertension is not only a key mediator in the relationship between TCBI and stroke, but also an important modulator that exacerbates the negative effects of metabolic dysregulation. In addition to hypertension, chronic inflammation (40, 41), coagulopathy, and insulin resistance also play critical roles in the association between TCBI and stroke. Elevated TCBI is often associated with chronic low-grade inflammation, particularly in individuals with obesity and metabolic syndrome (42, 43). This inflammation leads to an elevation of proinflammatory cytokines, such as interleukin-6 and C-reactive protein, which disrupt endothelial function and promote atherosclerosis (42, 43). Moreover, chronic inflammation can enhance platelet aggregation, increasing the likelihood of thrombus formation (44, 45). Elevated TCBI is also linked to hyperlipidemia and obesity, which can increase blood viscosity and promote platelet aggregation, further heightening the risk of thrombus formation (46, 47). When atherosclerotic plaques rupture, thrombus formation can block blood vessels, leading to stroke (48). Additionally, insulin resistance (IR), which is strongly linked to stroke risk (49–51), accelerates platelet aggregation, induces endothelial dysfunction, and promotes inflammatory responses—all of which contribute to an increased risk of stroke (52, 53). IR also disrupts cholesterol metabolism and accelerates vascular smooth muscle cell proliferation, further driving atherosclerosis and stroke progression (54).

The observed dose-response relationship provides quantitative reference values for the clinical prediction of stroke risk in middle-aged and older populations, reinforcing the clinical utility of TCBI as a predictive marker. Additionally, stratified analyses confirm the robustness of our findings, enhancing the reliability of the observed associations. Furthermore, we found hypertension as a mediator in the relationship between TCBI and stroke occurrence. This finding offers new insights into the underlying mechanisms linking TCBI to stroke risk and highlights the importance of early intervention and comprehensive risk management strategies.

Despite its strengths, this study has several limitations. First, while our findings suggest a significant association between TCBI and stroke risk, causality cannot be definitively established in this prospective cohort study. To clarify this relationship, future research should utilize more robust causal inference methods, such as randomized controlled trials or Mendelian randomization. Additionally, while the study design reduces the risk of reverse causality, there remains a possibility that preclinical stroke could influence body weight and lipid levels, which may affect the observed associations. Second, stroke diagnoses were based on self-reported physician assessments without confirmatory imaging (e.g., CT or MRI) or ICD codes, which may introduce information bias. Confirmatory imaging should be incorporated in future studies to enhance diagnostic accuracy. Third, although we adjusted for several confounders, factors such as inflammatory biomarkers, dietary habits, exercise, and genetic predispositions were not included and may influence the observed associations. Incorporating these factors into future research would provide a more comprehensive understanding of the relationship between TCBI and stroke. Additionally, only two exposure measurements were available in this study, and lipid and weight trajectories between these measurements were not tracked. This limitation may affect the accuracy of the cumulative exposure estimates and the interpretation of the findings. Fourth, since this study was conducted in a middle-aged and older Chinese population, the findings may not be directly generalizable to other ethnic groups or younger populations. Further research in diverse populations is needed to assess the broader applicability of these results. Lastly, while we used Bootstrap to assess the stability of the mediating effect, further studies with larger sample sizes and more diverse cohorts are needed to validate the robustness and clinical significance of this mediating effect. Moreover, exploring the underlying biological mechanisms of this relationship is crucial for better understanding the role of TCBI and hypertension in stroke risk.

## 5 Conclusion

Our study demonstrated that CumTCBI is significantly associated with an increased risk of stroke, with hypertension playing a mediating role in this relationship, underscoring the importance of blood pressure management in stroke prevention. These findings provide strong evidence to address existing research gaps and support the potential of TCBI as a simple and effective biomarker for identifying individuals at high risk of stroke. Regular monitoring and management of TG, TC, BW, and blood pressure may contribute to reducing stroke risk in middle-aged and older populations.

## Data Availability

The datasets presented in this study can be found in online repositories. The names of the repository/repositories and accession number(s) can be found at: CHARLS datasets are available for download at the CHARLS home website (http://charls.pku.edu.cn/en).

## Footnotes

1
http://www.R-project.org
