# Supplementary material for: Unraveling the role of cumulative triglyceride-total cholesterol-body weight index in stroke development: evidence from the CHARLS cohort
Source: Front Med (Lausanne). 2025 Jul 10;12:1616520. doi: 10.3389/fmed.2025.1616520 (PMC12287075; doi:10.3389/fmed.2025.1616520)
Supplement: Supplementary file 1 [file Table_1.docx]

| Supplementary Table 1. Details of missing values. | | | |
| --- | --- | --- | --- |
| **Variable** | **Valid Samples** | **Missing Samples** | **Missing Proportion** |
| Education level | 5595 | 3 | 0.05% |
| Drinking | 5595 | 3 | 0.05% |
| Hypertension | 5585 | 13 | 0.23% |
| Diabetes | 5564 | 34 | 0.61% |
| Heart disease | 5582 | 16 | 0.29% |
| Dyslipidemia | 5490 | 108 | 1.97% |
| Liver disease | 5572 | 26 | 0.47% |
| Kidney disease | 5575 | 23 | 0.41% |
| CumFBG | 5593 | 5 | 0.09% |
| CumHbA1c | 5561 | 37 | 0.67% |
| CumHDL-C | 5597 | 1 | 0.02% |
| CumLDL-C | 5586 | 12 | 0.21% |
| CumScr | 5591 | 7 | 0.13% |
| CumUA | 5597 | 1 | 0.02% |
| CumFBG, cumulative fasting blood glucose; CumHbA1c, cumulative glycosylated hemoglobin A1c; CumHDL‐c, cumulative high‐density lipoprotein cholesterol; CumLDL-c, cumulative low-density lipoprotein cholesterol; CumSCr, cumulative serum Creatinine; CumUA, cumulative uric acid. | | | |

| Supplementary Table 2. Baseline characteristics of individuals classified by the outcome. | | | |
| --- | --- | --- | --- |
| **Characteristics** | **Non - stroke** | **Stroke** | **P-value** |
| n (%) | 5098 (91.07%) | 500 (8.93%) |  |
| Sex (Female), n (%) | 2813 (55.2) | 283 (56.6) | 0.542 |
| Age, years | 58.5 ± 8.5 | 60.8 ± 8.1 | < 0.001 |
| Residence (Urban) | 1680 (33) | 149 (29.8) | 0.151 |
| Education |  |  | 0.297 |
| Elementary school or below, n (%) | 2428 (47.7) | 255 (51) |  |
| Middle school, n (%) | 2188 (42.9) | 205 (41) |  |
| College or above, n (%) | 479 (9.4) | 40 (8) |  |
| Current married, n (%) | 4580 (89.8) | 435 (87) | 0.047 |
| SBP, mmHg | 127.7 ± 20.4 | 134.9 ± 22.8 | < 0.001 |
| DBP, mmHg | 74.7 ± 11.9 | 77.8 ± 12.5 | < 0.001 |
| CumBMI, kg/m2 | 23.2 (21.0, 25.8) | 24.2 (21.8, 26.9) | < 0.001 |
| Smoking |  |  | 0.050 |
| Never, n (%) | 3187 (62.5) | 302 (60.4) |  |
| Former, n (%) | 392 (7.7) | 54 (10.8) |  |
| Current, n (%) | 1519 (29.8) | 144 (28.8) |  |
| Drinking |  |  | 0.103 |
| Never, n (%) | 3031 (59.5) | 294 (58.9) |  |
| Former, n (%) | 379 (7.4) | 50 (10) |  |
| Current, n (%) | 1686 (33.1) | 155 (31.1) |  |
| **Comorbidities** |  |  |  |
| Dyslipidemia, n (%) | 419 (8.4) | 92 (18.6) | <0.001 |
| Hypertension, n (%) | 2267 (44.6) | 315 (63) | <0.001 |
| Heart disease, n (%) | 524 (10.3) | 93 (18.6) | <0.001 |
| Diabetes, n (%) | 685 (13.5) | 94 (18.8) | 0.001 |
| Liver disease, n (%) | 158 (3.1) | 17 (3.4) | 0.714 |
| Kidney disease, n (%) | 270 (5.3) | 32 (6.4) | 0.303 |
| **Laboratory parameters** | |  |  |
| CumFBG, mg/dL | 106.0 ± 28.9 | 112.3 ± 39.2 | <0.001 |
| CumHbA1c, % | 5.6 ± 0.8 | 5.8 ± 1.0 | < 0.001 |
| CumTG, mg/dL | 105.3 (74.3, 154.0) | 115.5 (84.1, 169.0) | < 0.001 |
| CumTC, mg/dL | 193.4 ± 38.6 | 198.5 ± 37.8 | 0.005 |
| CumHDL-C, mg/dL | 51.6 ± 12.5 | 49.6 ± 12.1 | < 0.001 |
| CumLDL-C, mg/dL | 109.7 ± 28.5 | 112.7 ± 30.0 | 0.026 |
| CumScr, mg/dL | 0.8 ± 0.2 | 0.8 ± 0.2 | 0.007 |
| CumUA, mg/dL | 4.4 ± 1.2 | 4.5 ± 1.3 | 0.037 |
| CumTCBI | 1243.9  (817.1, 1996.9) | 1473.6  (984.1, 2206.7) | < 0.001 |
| Note: Variables are presented as mean ± SD, median (IQR1,3) or n (%).  SBP, systolic blood pressure; DBP, diastolic blood pressure; CumBMI, cumulative body mass index; CumFBG, cumulative fasting blood glucose; CumHbA1c, cumulative glycosylated hemoglobin A1c; CumTG, cumulative triglyceride; CumTC,cumulative total cholesterol; CumHDL‐c, cumulative high‐density lipoprotein cholesterol; CumLDL-c, cumulative low-density lipoprotein cholesterol; CumSCr, cumulative serum Creatinine; CumUA, cumulative uric acid; CumTCBI, cumulative triglyceride Cholesterol Body-weight Index. | | | |

| Supplementary Table 3. Association between the CumTCBI and the risk of stroke after excluding individuals with missing values. | | | | | | | |
| --- | --- | --- | --- | --- | --- | --- | --- |
| **CumTCBI** | | **Quartiles** | | | | | **Continuous** |
|  |  | **Q1** | **Q2** | **Q3** | **Q4** | **P for trend** | **Per 1 SD increase** |
| Median | | 637.4 | 1030.6 | 1573.3 | 2797.4 |  |  |
| Cases, n (%) | | 83 (6.1%) | 120 (8.9%) | 138 (10.3%) | 150 (11.2%) | – | – |
| Crude, HR (95% CI) | | Reference | 1.469  (1.11, 1.943) | 1.702  (1.297, 2.235) | 1.902  (1.455, 2.487) | < 0.001 | 1.213  (1.123, 1.311) |
| Model 1, HR (95% CI) | | Reference | 1.52  (1.148, 2.011) | 1.809  (1.376, 2.38) | 2.096  (1.598, 2.75) | < 0.001 | 1.257  (1.164, 1.358) |
| Model 2, HR (95% CI) | | Reference | 1.529  (1.155, 2.025) | 1.82  (1.382, 2.396) | 2.115  (1.608, 2.781) | < 0.001 | 1.261  (1.166, 1.363) |
| Model 3, HR (95% CI) | | Reference | 1.397  (1.049, 1.86) | 1.549  (1.161, 2.067) | 1.647  (1.224, 2.218) | 0.001 | 1.167  (1.064, 1.279) |
| Crude model adjusts for: none; | | | | | | | |
| Model I adjust for sex and age; | | | | | | | |
| Model II adjusts for Model I, marital status, residence, education, smoking, and drinking; | | | | | | | |
| Model III adjusts for Model II, hypertension, heart disease, diabetes, liver disease, kidney disease, CumFBG, CumHbA1c, CumLDL-C, CumScr, CumUA. | | | | | | | |
| CI, confidence interval; HR, hazard ratio. | | | | | | | |

| Supplementary Table 4. Baseline characteristics before and after 1:1 propensity score matching. | | | | | | | |
| --- | --- | --- | --- | --- | --- | --- | --- |
| **Covariates** | **Before Matching** | | |  | **After Matching** | | |
|  | **Q1-Q2** | **Q3-Q4** | **SMD** |  | **Q1-Q2** | **Q3-Q4** | **SMD** |
| n | 2799 | 2799 |  |  | 1703 | 1703 |  |
| Gender (Female) | 1429 (51.05) | 1667 (59.56) | 0.172 |  | 968 (56.84) | 984 (57.78) | 0.019 |
| Age, years | 59.64 ± 8.81 | 57.70 ± 8.10 | 0.224 |  | 58.27 ± 8.30 | 58.53 ± 8.40 | 0.031 |
| Residence (Urban) | 2013 (71.92) | 1756 (62.74) | 0.197 |  | 1150 (67.53) | 1143 (67.12) | 0.009 |
| Education level |  |  |  |  |  |  |  |
| Elementary school or below | 1428 (51.05) | 1255 (44.85) | 0.124 |  | 787 (46.21) | 790 (46.39) | 0.004 |
| Middle school | 1136 (40.61) | 1257 (44.92) | 0.087 |  | 751 (44.1) | 749 (43.98) | 0.002 |
| College or above | 233 (8.33) | 286 (10.22) | 0.066 |  | 165 (9.69) | 164 (9.63) | 0.002 |
| Current married | 2456 (87.75) | 2559 (91.43) | 0.121 |  | 1549 (90.96) | 1543 (90.6) | 0.012 |
| SBP, mmHg | 125.61 ± 20.34 | 131.03 ± 20.81 | 0.263 |  | 127.50 ± 20.98 | 129.01 ± 20.83 | 0.072 |
| DBP, mmHg | 72.92 ± 11.49 | 77.08 ± 12.14 | 0.356 |  | 74.13 ± 11.81 | 74.78 ± 11.89 | 0.055 |
| Smoking status |  |  |  |  |  |  |  |
| Never | 1658 (59.24) | 1831 (65.42) | 0.128 |  | 1103 (64.77) | 1099 (64.53) | 0.005 |
| Former | 203 (7.25) | 243 (8.68) | 0.052 |  | 124 (7.28) | 126 (7.4) | 0.005 |
| Current | 938 (33.51) | 725 (25.9) | 0.167 |  | 476 (27.95) | 478 (28.07) | 0.003 |
| Drinking status |  |  |  |  |  |  |  |
| Never | 1609 (57.53) | 1716 (61.33) | 0.077 |  | 1028 (60.36) | 1050 (61.66) | 0.027 |
| Former | 209 (7.47) | 220 (7.86) | 0.015 |  | 128 (7.52) | 120 (7.05) | 0.018 |
| Current | 979 (35) | 862 (30.81) | 0.089 |  | 547 (32.12) | 533 (31.3) | 0.018 |
| Dyslipidemia | 133 (4.83) | 378 (13.82) | 0.314 |  | 118 (6.93) | 131 (7.69) | 0.029 |
| Hypertension | 1073 (38.43) | 1509 (54.03) | 0.317 |  | 778 (45.68) | 771 (45.27) | 0.008 |
| Heart disease | 239 (8.56) | 378 (13.55) | 0.160 |  | 148 (8.7) | 155 (9.12) | 0.015 |
| Diabetes | 259 (9.29) | 520 (18.73) | 0.275 |  | 195 (11.45) | 224 (13.15) | 0.052 |
| Liver disease | 99 (3.55) | 76 (2.73) | 0.047 |  | 55 (3.23) | 47 (2.76) | 0.028 |
| Kidney disease | 155 (5.56) | 147 (5.28) | 0.012 |  | 102 (5.99) | 97 (5.7) | 0.012 |
| FBG, mg/dL | 100.84 ± 23.76 | 112.30 ± 34.20 | 0.389 |  | 103.58 ± 27.81 | 106.02 ± 23.82 | 0.094 |
| HbA1c, % | 5.50 ± 0.65 | 5.77 ± 0.93 | 0.337 |  | 5.56 ± 0.75 | 5.62 ± 0.70 | 0.083 |
| TG, mg/dL | 82.7 ± 29.19 | 186.71 ± 138.9 | 1.036 |  | 105.55 ± 30.35 | 108.17±28.90 | 0.088 |
| TC, mg/dL | 180.02 ± 32.10 | 207.71 ± 39.53 | 0.769 |  | 186.95 ± 31.99 | 189.65 ± 33.1 | 0.083 |
| HDL-c, mg/dL | 55.71 ± 12.79 | 47.09 ± 10.52 | 0.736 |  | 56.31 ± 12.79 | 55.3 ± 10.47 | 0.091 |
| LDL-c, mg/dL | 102.89 ± 23.85 | 117.08 ± 31.17 | 0.511 |  | 109.21 ± 23.46 | 109.16 ± 27.11 | 0.002 |
| CumScr, mg/dL | 0.76 ± 0.18 | 0.77 ± 0.18 | 0.056 |  | 0.77 ± 0.18 | 0.76 ± 0.17 | 0.057 |
| CumUA, mg/dL | 4.19 ± 1.15 | 4.54 ± 1.24 | 0.292 |  | 4.37 ± 1.19 | 4.29 ± 1.09 | 0.070 |

SBP, systolic blood pressure; DBP, diastolic blood pressure; CumBMI, cumulative body mass index; CumFBG, cumulative fasting blood glucose; CumHbA1c, cumulative glycosylated hemoglobin A1c; CumTG, cumulative triglyceride; CumTC,cumulative total cholesterol; CumHDL‐c, cumulative high‐density lipoprotein cholesterol; CumLDL-c, cumulative low-density lipoprotein cholesterol; CumSCr, cumulative serum Creatinine; CumUA, cumulative uric acid; CumTCBI, cumulative triglyceride Cholesterol Body-weight Index.
