# Supplementary figures and images for: Unraveling the role of cumulative triglyceride-total cholesterol-body weight index in stroke development: evidence from the CHARLS cohort
Source: Front Med (Lausanne). 2025 Jul 10;12:1616520. doi: 10.3389/fmed.2025.1616520 (PMC12287075; doi:10.3389/fmed.2025.1616520)

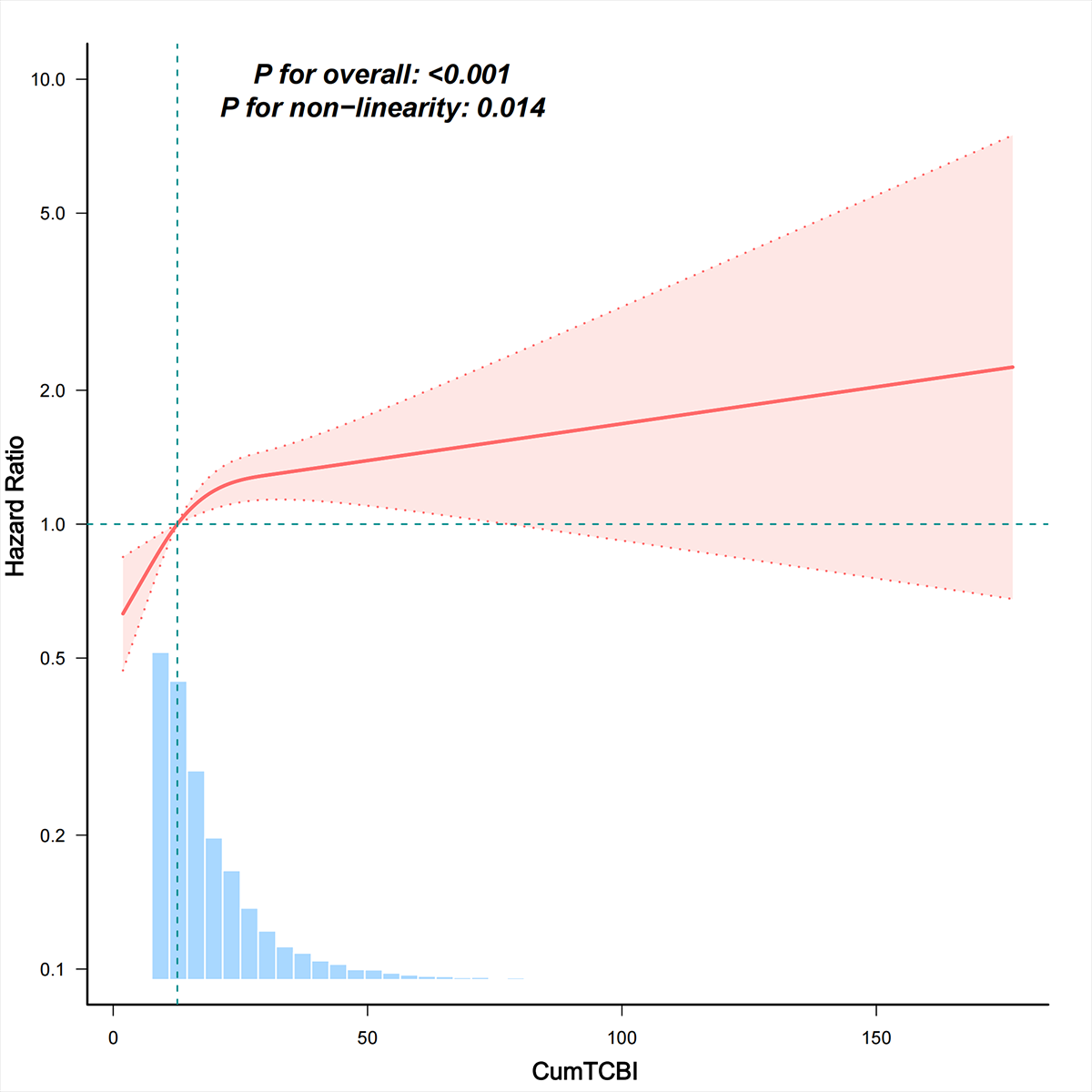

Supplement: Supplementary file 2 [file Image_1.tif]
